# Detection and Genetic Diversity of Heritable Bacterial Symbionts in Human Lice Based on 16S‐rRNA Gene

**DOI:** 10.1111/1758-2229.70243

**Published:** 2026-01-08

**Authors:** Anthony Marteau, Sophie Brun, Arezki Izri, Mohammad Akhoundi

**Affiliations:** ^1^ Parasitology‐Mycology Department Avicenne Hospital, AP‐HP, Sorbonne Paris Nord University Bobigny France; ^2^ Unité des Virus Émergents (UVE: Aix‐Marseille Univ, Università di Corsica) Marseille France

**Keywords:** *Candidatus* Riesia pediculicola, *Candidatus* Riesia pthiripubis, endosymbionts, human lice, *Wolbachia*

## Abstract

Human lice are obligate bloodsucking ectoparasites harbouring endosymbiotic bacteria essential for their survival. Despite the medical significance of human lice, their endosymbionts remain understudied, and knowledge about their species identity, prevalence and genetic diversity is largely limited. Head, body and pubic louse specimens' collection from infested patients of various origins between 2019 and 2023 enabled molecular screening for distribution and genetic diversity of bacterial endosymbionts through conventional PCR targeting two fragments of 16S‐rRNA. A total of 209 louse specimens isolated from infested patients, including 186 head lice, 11 body lice and 12 pubic lice were examined with 77.5%, 41.7% and 94.3% of the specimens found to be infested with *Candidatus* Riesia pediculicola, *Candidatus* Riesia pthiripubis and *Wolbachia* respectively. Inferred phylogenetic analysis of *Candidatus* Riesia and *Wolbachia* sequences revealed heterogeneity clustering into four and three clades respectively. No specific correlation was observed between these endosymbionts and lice ecological forms or geographical origin demonstrating that head, body and pubic lice share the same *Candidatus* Riesia and *Wolbachia* strains with independent adaptation and co‐evolution, except *Candidatus* Riesia pthiripubis which was identified exclusively in pubic lice. These phylogenetic results were aligned by network analysis. These findings could be helpful in evolutionary and biological control investigations.

## Graphical abstract

*Candidatus* Riesia pediculicola and *Wolbachia* were found in 79.6% and 95.2% of head lice, and 81.8% and 100% of body lice. *Candidatus* Riesia pthiripubis and *Wolbachia* appeared in 41.7% and 75% of pubic lice. Maximum‐likelihood 16S‐rRNA phylogeny revealed substantial heterogeneity within symbiont populations.

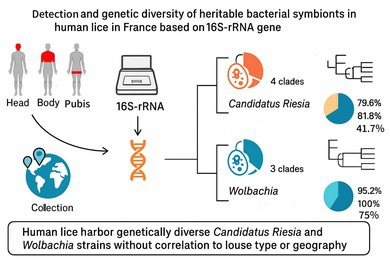

## 1 Introduction

Human lice are most likely amongst the oldest permanent ectoparasites of humans (Boutellis et al. 2014; Mumcuoglu 2008). They are distributed worldwide with more prevalence in tropical and subtropical countries. They feed on humans of all ages and sexes across all socioeconomic levels (Veracx and Raoult 2012). There are two species: (a) two ecological forms of the human louse ( *Pediculus humanus* ), the head louse ( *Pediculus humanus capitis* ), and the body louse ( *Pediculus humanus corporis* ); and (b) the pubic louse ( *Pthirus pubis* ) (Akhoundi et al. 2024).

Head lice (*P. h. capitis*) primarily affect school‐aged children, with prevalence rates ranging from 3% to 49% depending on region (Chosidow 2000; Rukke et al. 2011). They inhabit the scalp and lay eggs on hair shafts causing pruritus especially on the scalp, neck and shoulders (Chosidow 2000; Durand et al. 2018). Although suspected, their role in disease transmission remains unproven (Veracx and Raoult 2012). Body lice (*P. h. corporis*) is typically associated with poor hygiene and found in clothing and bedding (Amanzougaghene et al. 2020; Badiaga and Brouqui 2012). They are known vectors for pathogenic bacteria, such as *Rickettsia prowazekii* , etiologic agent of epidemic typhus, *Borrelia recurrentis* , causative agent of louse‐borne relapsing fever, and *Bartonella quintana* , responsible of trench fever (Fournier et al. 2002; Raoult et al. 1998; Sangaré et al. 2015). Pubic lice ( *Pthirus pubis* ) commonly infest pubic hair, and occasionally other body areas such as armpit hair, chest hair, eyelashes or scalp hair (Chosidow 2000). Whilst DNA of some pathogens has been detected in pubic lice, it is not recognised as vector of infectious diseases (Mana et al. 2017; Patel et al. 2021).

The life cycle of insects, like other organisms, is influenced by their microbiota. The relationship between insects and their microbiota is crucial to ensuring proper physiological function and health in insects. This evolutionary relationship between a host and its symbionts is a common phenomenon amongst arthropods including human lice (Arora and Douglas 2017; Sassera et al. 2013). The insects generally harbour endosymbionts that play various roles in their life cycle. Symbionts contribute to various processes including digestion and anabolic activities (supply of vitamins and amino acids) (Akman et al. 2002), protection against natural enemies (Gosalbes et al. 2010), stimulation of the host's natural immune defences (Kubiak et al. 2018), degradation of insecticides (Su et al. 2013) and competence of their host for transmitting pathogenic agents (Kliot and Ghanim 2013). The primary symbionts are specific to their host, providing it with essential functions. In contrast, the secondary symbionts can be found in different hosts (Perotti et al. 2007).

In blood‐sucking lice species of animals, various endosymbiotic bacteria with provisional *Candidatus* status have been described. These include species such as *Candidatus* Legionella polyplacis, *Candidatus* Riesia pediculschaeffi and *Candidatus* Riesia sp. GBBU which have been identified in lice collected from animals like mice, chimpanzees and gorillas, responsible for the synthesis of essential B‐vitamins (Boyd et al. 2017; Hypsa and Krizek 2007; Mahmood et al. 2023; Perotti et al. 2004; Ríhová et al. 2017). This species‐specific adaptation of *Candidatus* to various louse species parasitizing humans and other animals (Kubiak et al. 2018; Mahmood et al. 2023; Perotti et al. 2004; Ríhová et al. 2017), suggests the presence of this symbiont in the common ancestor of lice parasitizing humans, chimpanzees, and gorillas (Boyd et al. 2024).

In human lice – particularly in body and pubic lice – despite their medical importance, knowledge about microbial symbionts and their potential roles in louse biology is largely limited (Agany et al. 2020). Based on the literature, only three endosymbionts have been identified in human lice (Allen et al. 2007; Boyd et al. 2024). They include *Candidatus* Riesia pediculicola in head and body lice, *Candidatus* Riesia pthiripubis in pubic lice and *Wolbachia* in head, body and pubic lice (Allen et al. 2007; Kyei‐Poku et al. 2005; Perotti et al. 2008; Sasaki‐Fukatsu et al. 2006).

*Candidatus* Riesia pediculicola is an obligate Gram‐negative intracellular bacterium belonging to the family Enterobacteriaceae (Allen et al. 2007; Perotti et al. 2007). This bacterium resides in the lice bacteriome, localised on the ventral side of the lice midgut and transmitted transovarially to progeny (Sasaki‐Fukatsu et al. 2006). It plays a crucial role in synthesising group B vitamins (B1, B2, B3, B5, B6, B7 and B9), which are vital for the lice but absent in their human blood meal (Burkhart and Burkhart 2006; Puchta 1955). For instance, pantothenic acid (vitamin B5) is important for the growth of the louse and its absence leads to the death of the nymph during the first moult (Perotti et al. 2009). It possesses several specific genes encoding transport and binding proteins and enzymes involved in lipopolysaccharide biosynthesis. Due to the enzymes responsible for energy metabolism, it depends on the louse for nutrients. It has been associated with its louse hosts for 13–25 million years indicating its role in the adaptation and co‐evolution processes alongside its louse host (Allen et al. 2009; Hammoud et al. 2022). *Candidatus* Riesia pthiripubis is the endosymbiont of the same family detected in pubic lice (Allen et al. 2007; Boyd et al. 2024). Similar to *Candidatus* Riesia pediculicola, this bacterium fulfils a comparable role in supplementing the louse's blood‐based diet, which lacks certain essential nutrients. By supplying these missing nutrients, *Candidatus* Riesia pthiripubis enables the louse to thrive on its specialised diet. A long‐term co‐evolutionary relationship exists between pubic lice and *Candidatus* Riesia pthiripubis, indicating that the bacterium has evolved alongside its louse host over extended periods (Allen et al. 2007).

*Wolbachia* is another symbiont widely distributed component of many arthropods including human lice, in which it is a secondary symbiont. It is a Gram‐negative α‐proteobacterium, closely related to the *Rickettsia* species. These intracellular bacteria are transmitted vertically and occasionally horizontally and affect various host reproductive processes (e.g., cytoplasmic incompatibility, feminisation, parthenogenesis, increased or decreased fitness, and obligate symbiosis) (Girin and Bouletreau 1995; Hurst et al. 1999; Min and Benzer 1997; O'Neill et al. 1992; Rousset et al. 1992). Despite this affection, in some host species where an obligate or mutualistic relationship has developed, elimination of *Wolbachia* symbionts results in retarded growth and sterility of the insect host. They express a high genetic diversity, likely due to their interactions with many invertebrate hosts (O'Neill et al. 1992; Zhou et al. 1998). However, *Wolbachia* in human lice has been understudied with research limited to only a few studies (Kyei‐Poku et al. 2005; Perotti et al. 2008).

Despite the medical significance of human lice and the crucial role of its symbionts in metabolism and evolution, the distribution and genetic diversity of these endosymbionts in relation to louse host species are not comprehensively understood (Boyd et al. 2024). In this study, we aimed to evaluate the frequency and genetic diversity of the commonly reported endosymbiotic bacteria, *Candidatus* Riesia and *Wolbachia*, within louse populations of the patients with different geographical origins inhabiting the northern suburbs of Paris.

## 2 Materials and Methods

### 2.1 Human Louse Collection

Human louse specimens were obtained by parasitological examination of suspected patients referred to the Parasitology Department of the Avicenne Hospital (Bobigny, France) from March 2019 to March 2023. Head lice were gathered by combing the hair of infested patients. For body lice, the cloths of suspected individuals were subjected to parasitological examinations, whilst the pubic area of possibly infested individuals was examined clinically. The collected louse specimens were placed in sterile Petri dishes and examined morphologically under a stereomicroscope (Stemi 508, Carl Zeiss, Germany). Adult specimens of head, body and pubic lice were identified separately according to the morphological identification key of Ewing (1926, 1929).

### 2.2 DNA Extraction, PCR Amplification and Species Assignation

The collected louse DNA was extracted individually using Chelex 10% (Bio‐Rad, CA, USA) (Walsh et al. 1991). The processed specimens underwent screening and symbiont species identification by conventional PCR targeting two different fragments of the 16S‐rRNA gene (Weisburg et al. 1991; Werren and Windsor 2000). In brief, each PCR reaction was performed in a final volume of 25 μL, including 12.5 μL master mix (AmpliTaq Gold 360 Master Mix, Applied Biosystems), 8 μL distilled water, 0.75 μL of forward and reverse primers for each fragment amplification and finally 3 μL extracted template DNA. The first PCR batch for *Candidatus* Riesia screening was performed by amplification of 1500 bp of 16S‐rRNA using forward (16suF: 5′‐GAGTTTGATCCTGGCTCAG‐3′) and reverse (16suR: 5′‐GTTACCTTGTTACGACTT‐3′) primers (Weisburg et al. 1991). The PCR reactions were performed under the following conditions: initial denaturation at 95°C for 5 min, followed by 35 cycles of 95°C for 1 min, annealing at 55°C for 1 min, 72°C for 1 min and a final extension at 72°C for 5 min. The second PCR of the 16S‐rRNA gene for *Wolbachia* screening was performed using forward (16S W‐Spec F: 5′‐CATACCTATTCGAAGGGATAG‐3′) and reverse (16S W‐Spec R: 5′‐AGCTTCGAGTGAAACCAATTC‐3′) primers, allowing the amplification of a fragment of 438 base pairs (Werren and Windsor 2000). The PCR program was carried out with an initial denaturation at 95°C for 5 min, followed by 40 cycles of 95°C for 30 s, 60°C for 1 min, 72°C for 45 s, with a final extension of 72°C for 5 min. Negative and positive controls were included in each PCR batch. The amplicons were analysed using electrophoresis on a 1.5% agarose gel containing ethidium bromide.

### 2.3 Genetic Diversity Analysis and Phylogenetic Reconstruction

PCR products were then subjected to bidirectional DNA sequencing with the same primer pairs used for amplification. The sequences were edited, aligned and compared with homologous sequences from GenBank using the BLAST (Basic Local Alignment Search Tool) (www.ncbi.nlm.nih.gov/BLAST). Strains were assigned to species level, based on ≥ 99% homology to GenBank sequences. Sequence alignment was performed with the BioEdit v7.0.0 software (Hall 1999), and the phylogenetic analysis was carried out using MEGA v.11 software (Kumar et al. 2004). The inferred phylogenetic trees of *Candidatus* Riesia and *Wolbachia* species (identified in this study) and homonym sequences from GenBank were constructed using the maximum likelihood (ML) method under the Tamura–Nei model. Bootstrap values were determined by 1000 replicates. The best‐fit substitution model was determined using MEGA, and the nearest‐neighbour‐interchange (NNI) algorithm was employed as the ML heuristic method. To display the genetic relationships within *Candidatus* Riesia and *Wolbachia* populations, the median‐joining algorithms were implemented using NETWORK v.5 software (Bandelt et al. 1999). Haplotype frequency for *Candidatus* Riesia and *Wolbachia* populations was calculated by DnaSP v6.12.03 (Rozas et al. 2003). The genetic distance of processed louse specimens is provided in SI.IV in Supporting Information.

## 3 Results

A total of 209 patients (157 women and 52 men) were examined and found to be infested with human lice. They included 186 patients with head lice, 11 with body lice and 12 with pubic lice. A little less than half of the examined patients (44.5%) were French, whilst the remaining patients originated from 17 countries: Asia (Bangladesh, India, Lebanon, Pakistan and Sri Lanka), Africa (Algeria, Gabon, Ivory Coast, Mauritius, Morocco, Togo and Tunisia), South America (Venezuela) and Europe (Italy, Portugal, Romania and Serbia). One louse specimen, randomly selected per patient, was analysed using conventional PCRs targeting two fragments of the 16S‐rRNA gene. Detailed information of the processed patients, collected louse specimens, symbiont species identified and their positivity rate is provided in Table 1.

**TABLE 1:** Detailed information of the human louse specimens examined for bacterial symbiont screening in this study.

Lice species	Number of infested patients examined		Origin^a^	Number of louse specimens examined	Number and positivity rate (%) of bacterial symbionts identified		
	Man	Woman			*Candidatus* Riesia pediculicola	*Candidatus* Riesia pthiripubis	*Wolbachia*
*Pediculus humanus capitis*	33	153	14	186	148 (79.6%)	—	177 (95.2%)
*Pediculus humanus corporis*	9	2	7	11	9 (81.8%)	—	11 (100%)
*Pthirus pubis*	10	2	5	12	—	5 (41.7%)	9 (75%)
Total	52	157	18	209	157 (79.7%)	5 (41.7%)	197 (94.3%)

^a^ Number of countries to which the patients belong.

**FIGURE 1:**
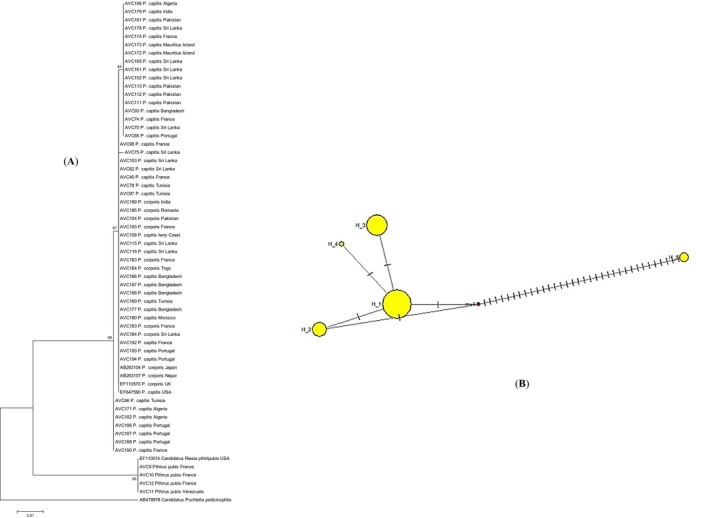
(A) Maximum likelihood (ML) tree reconstructed from the 16S‐rDNA sequences of *Candidatus* isolates detected in human louse specimens of various origin (beginning with AVC) and sequences from GenBank. (B) Median‐joining network for the *Candidatus* 16S‐rDNA sequences of human louse specimens processed in this study.

The screening PCR targeting the first fragment for detection of *Candidatus* Riesia endosymbionts revealed positivity in 77.5% of the analysed specimens (162 out of 209 louse specimens). They included 79.6% (148/186) head lice, 81.8% (9/11) body lice and 41.7% (5/12) pubic lice. Amongst them, 110 sequences with adequate quality representing the louse species and geographical locality, were selected and subjected to phylogenetic analyses. The obtained sequences were deposited in GenBank under the following accession numbers: *Candidatus* Riesia (PX057027–PX057077) and *Wolbachia* (PX059384–PX059444) (Table SI.1 in Supporting Information). The inferred phylogenetic tree (ML), demonstrated a heterogeneity, with the analysed sequences clustering into four clades (Figure 1A). Whilst the first three clades were composed of *Candidatus* Riesia pediculicola identified in head and body louse specimens from various countries, the fourth clade included exclusively the *Candidatus* Riesia pthiripubis determined in pubic louse samples from France, Venezuela and United States.

**FIGURE 2:**
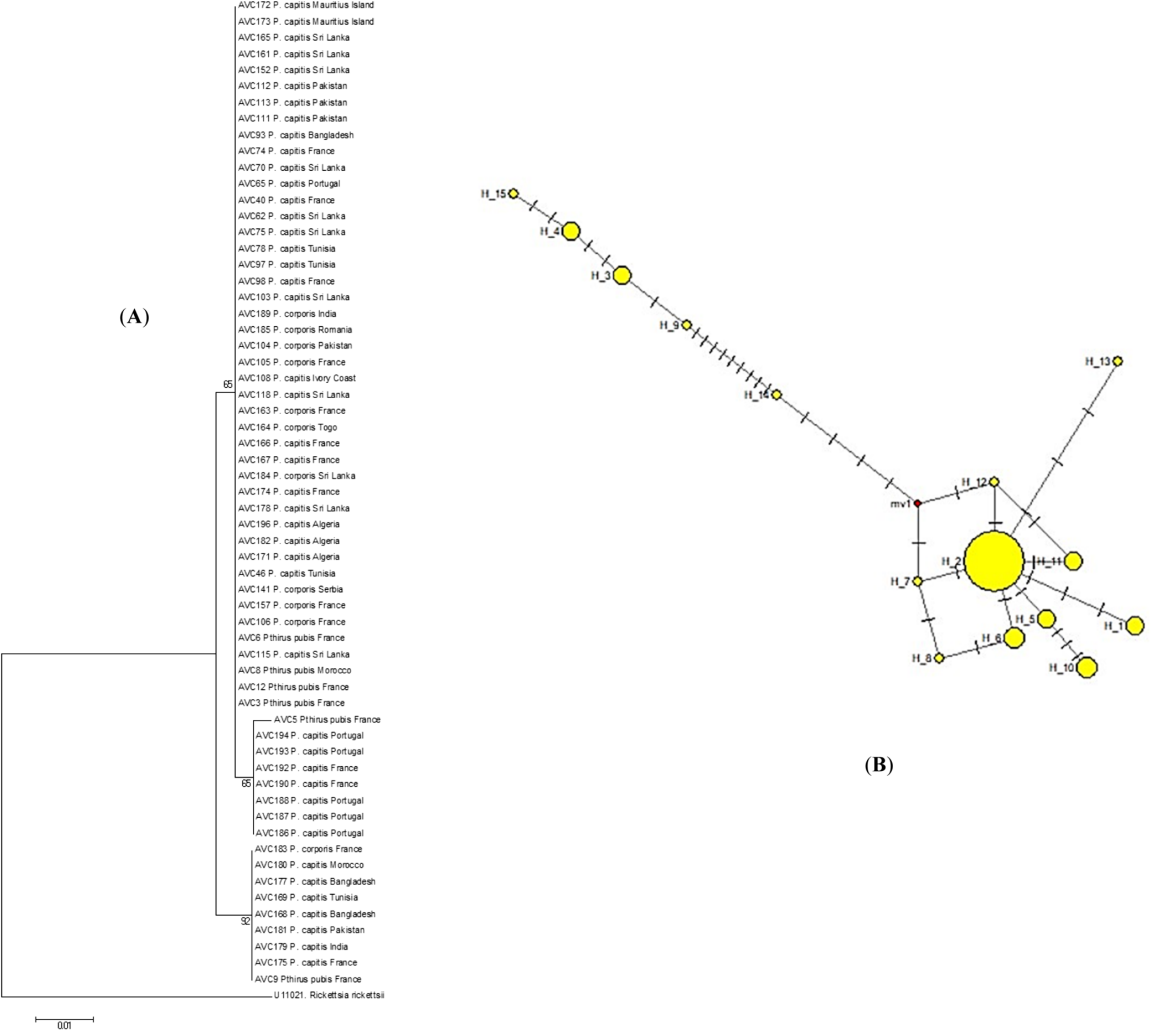
(A) ML tree reconstructed using *Wolbachia* 16S‐rDNA sequences identified in human louse specimens of various origin. (B) Median‐joining network representing the 16S‐rDNA sequences of *Wolbachia* in human louse specimens.

The screening PCR targeting the second fragment for *Wolbachia* detection showed 94.3% (197/209 lice) of specimens infested, including 95.2% (177/186) head lice, 100% (11/11) body lice and 75% (9/12) pubic lice. Then, 121 sequences, suitable for analysis and representing the louse species and geographical origins, were chosen to construct the phylogenetic tree using the ML method. The resulting phylogenetic tree demonstrated heterogeneity with sequences grouped in three clades: the first clade, included *Wolbachia* specimens detected in head, body and pubic lice from Asia, Africa and Europe; the second clade was comprised of *Wolbachia* in head and pubic lice with a European origin, whilst the third one was composed of *Wolbachia* detected in all human louse specimens from Asian, African and European origins (Figure 2A).

A similar finding was observed through network analysis, consistent with the results of the phylogenetic analyses using the ML method (Figures 1B and 2B). Haplotype frequency for *Candidatus* Riesia and *Wolbachia* populations is provided in SI.II and SI.III in Supporting Information. Moreover, the estimated evolutionary divergence within *Candidatus* Riesia and *Wolbachia* specimens is provided in SI.IV in Supporting Information.

**FIGURE 3:**
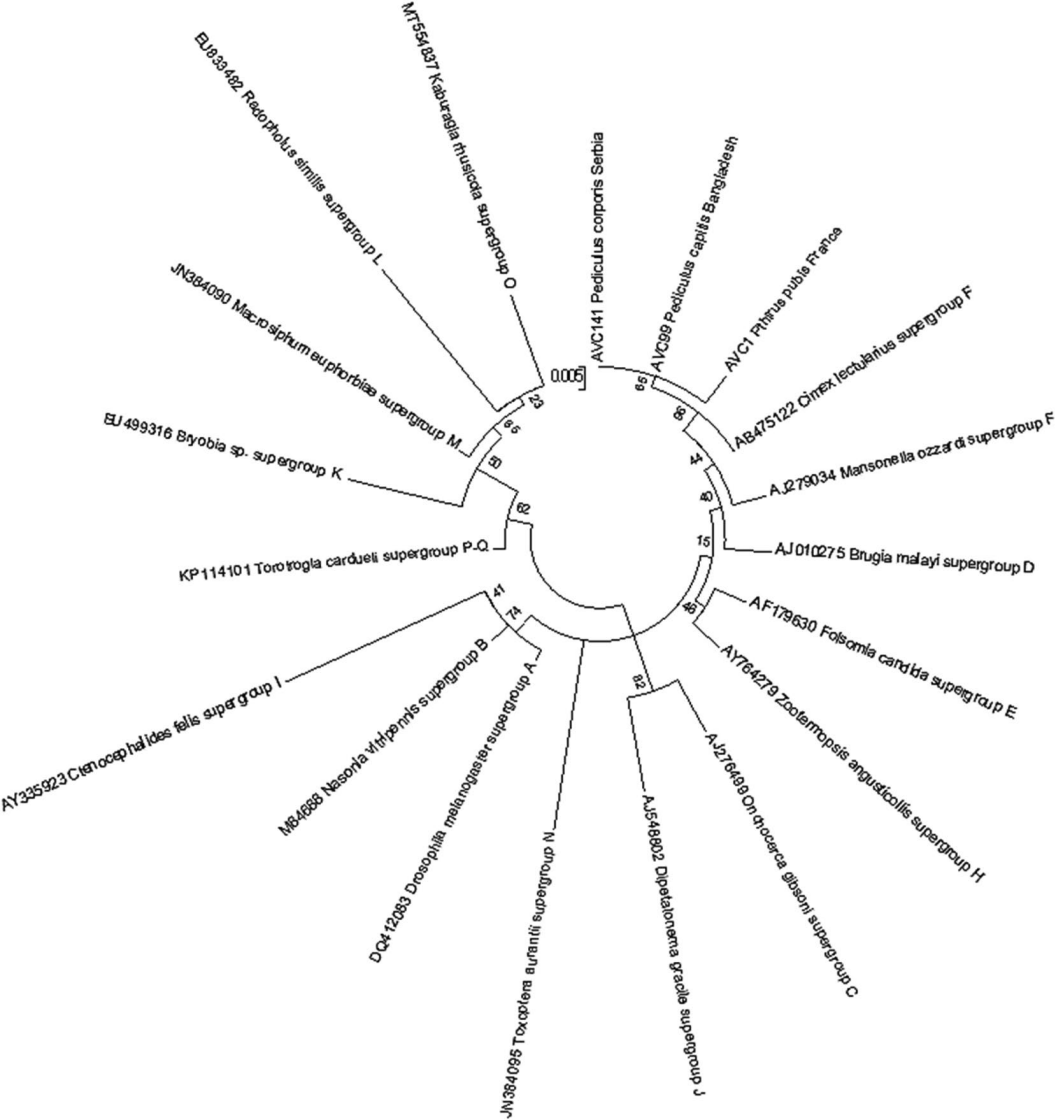
Unrooted phylogenetic tree of *Wolbachia* 16S‐rDNA sequences belonging to head, body and pubic lice specimens we collected (named AVC) and *Wolbachia* strains reported from diverse arthropod and helminth hosts collected from GenBank.

The phylogenetic analysis of *Wolbachia* strains identified in head, body and pubic lice in this study along with all known supergroups of *Wolbachia*, revealed that this human louse endosymbiont belongs to supergroup F (Figure 3).

## 4 Discussion

*Candidatus* Riesia pediculicola, *Candidatus* Riesia pthiripubis and *Wolbachia* are bacteria that exhibit an obligate presence in their louse hosts. They are the only endosymbionts commonly reported in human lice. Whilst most studies have focused on these endosymbionts in head and body lice (Allen et al. 2007; Sasaki‐Fukatsu et al. 2006), reports of *Candidatus* Riesia pthiripubis and *Wolbachia* in pubic lice remain limited to only a few reports (Kyei‐Poku et al. 2005; Perotti et al. 2008).

A summarised overview of the bacterial endosymbionts of human lice reported in the literature is provided in SI.V in Supporting Information. In the only study evaluating the positivity rate of *Wolbachia* amongst head louse specimens in the Yucatan region of Mexico, 71.42% of processed specimens were found to be infested (Dzul‐Rosado et al. 2022). However, this positivity rate was not replicated in other investigations, which only reported the *Wolbachia* detection (Covacin and Barker 2007; Kyei‐Poku et al. 2005). Similarly, a positivity rate of *Candidatus* Riesia ranging from 53.6% to 100% in head and body lice was reported in only a few investigations targeting ftsZ, groEL, rpoB and 16S‐rDNA genes (Boyd et al. 2024; Hammoud et al. 2022; Sasaki‐Fukatsu et al. 2006), whilst other researches did not specify the positivity rate (Allen et al. 2007). In this study, the high frequencies of *Candidatus* Riesia (77.5%) and *Wolbachia* (94.3%) confirm the endosymbiotic nature of these bacteria. However, we did not observe a 100% frequency of *Candidatus* Riesia and *Wolbachia* endosymbionts. Similarly, earlier studies reported the positivity rates ranging from 53.6% to 89% for *Candidatus* Riesia and 71.4% for *Wolbachia* (Boyd et al. 2024; Dzul‐Rosado et al. 2022; Hammoud et al. 2022). These variations may be attributed to physiological and biological factors, PCR amplification efficacy or other host‐related influences that remain poorly understood. Whilst most previous investigations have been limited by a small number of analysed specimens, focused on a single species of human lice, or restricted sampling locations (SI.III in Supporting Information), this study analysed 209 specimens across louse species – head, body, and pubic lice – collected from patients representing 18 countries. The latter allowed us to evaluate whether there is a correlation between *Candidatus* Riesia and *Wolbachia* endosymbionts and the louse species originating from various geographical regions. We were unable to trace the exact source of lice infestation in the patients examined in this study. The only confirmed information pertains to the origin of the infested patients themselves, not the origin of their lice infestation. However, most immigrant patients reported having been infested in their home countries (Table 1). This broader number of louse species and specimens examined allowed for a large‐scale assessment of the frequency of the mentioned endosymbiont species.

Phylogenetic analysis of *Candidatus* Riesia pediculicola identified in the head and body louse specimens in this study revealed heterogeneity, clustering into three distinct clades. Within *P. humanus* , no clear correlation was observed between *Candidatus* Riesia pediculicola sequences and the ecological form (head vs. body lice) or geographical origin, as similar symbiont haplotypes were distributed across human lice collected from various regions (Figure 1A,B). These findings confirm that head and body lice share the same primary endosymbiotic bacterium, *Candidatus* Riesia pediculicola (Boyd and Reed 2012). This genetic variation of *Candidatus* Riesia pediculicola, based on the analysis of housekeeping genes, has been shown to correlate with COI diversity in human lice (Hammoud et al. 2022), as both *Candidatus* Riesia pediculicola and the COI mtDNA gene are maternally inherited through transovarial transmission, highlighting their potential role in co‐evolution with their louse hosts. *Candidatus* Riesia pthiripubis, a cluster of coevolving *Candidatus* Riesia system associated with pubic lice was grouped exclusively in a distinct single clade.

*Wolbachia* endosymbionts exhibit high genetic diversity and have been classified into 18 phylogenetic groups or supergroups (A to T), depending on the arthropod or helminth host species (Chebbah et al. 2023). However, little is known about the distribution and genetic variation of *Wolbachia* in human lice (Covacin and Barker 2007; Kyei‐Poku et al. 2005). The phylogenetic analysis of this endosymbiont in head, body and pubic lice conducted in this study along with homologous sequences from GenBank revealed a heterogeneity in which *Wolbachia* sequences of head, body and pubic lice were divided into three clades, with no specific correlation with louse species or the geographical origin of the examined specimens. On the other hand, head, body and pubic lice share the same *Wolbachia* strains with various geographical origins (Figure 2A,B). In addition, no association between *Candidatus* Riesia and *Wolbachia* strains of the same louse specimen was observed demonstrating independent adaptation and co‐evolution of these endosymbionts with their louse hosts. Furthermore, *Wolbachia* endosymbionts in human lice have been demonstrated to belong to various supergroups. Whilst American (USA and Canada) and European (Italy) strains of human lice *Wolbachia* analysed using *Wolbachia* surface protein (*wsp*) have been reported to belong to A and B groups (Kyei‐Poku et al. 2005; Perotti et al. 2004, 2008), Australian domestic pigeons' *Wolbachia* strains were in a distinct group of F using SSU‐rRNA (Covacin and Barker 2007). Phylogenetic analysis including all known *Wolbachia* supergroups and the *Wolbachia* sequences of head, body and pubic lice targeting 16S‐rRNA in this study revealed their position in group F (Figure 3). Finally, based on BLAST and phylogenetic analysis, no genetic hybrids within *Candidatus* Riesia pediculicola and the *Wolbachia* specimens studied were observed.

The contrasting evolutionary patterns of *Candidatus* Riesia and *Wolbachia* are likely rooted in their distinct biological roles, ecological characteristics and transmission dynamics. *Candidatus* Riesia is a primary obligate endosymbiont of human lice, exhibiting a long‐term co‐evolutionary relationship driven by strict vertical transmission and high host specificity. This close association leads to co‐speciation and congruent evolutionary histories with the host, a pattern commonly observed in obligate mutualists (Boyd et al. 2014; Kirkness et al. 2010). In contrast, *Wolbachia* is a facultative endosymbiont capable of both vertical and horizontal transmission. It infects a wide array of arthropods and nematodes and can manipulate host reproduction to enhance its transmission. These features result in a highly dynamic evolutionary history, often marked by frequent host shifts and incongruent phylogenies relative to their hosts (Baldo et al. 2006; Gerth et al. 2014; Werren et al. 2008). These fundamental differences in host interaction and transmission strategy underlie their differing evolutionary histories.

## 5 Conclusion

We highlight the frequency of *Candidatus* Riesia pediculicola and *Wolbachia* in processed specimens with geographically diverse origins of head (79.6% and 95.2%) and body lice (81.8% and 100%) as well as *Candidatus* Riesia pthiripubis and *Wolbachia* in pubic lice (41.7% and 75%) respectively. A significant heterogeneity was observed within *Candidatus* Riesia and *Wolbachia* populations. No significant correlation was found between *Candidatus* Riesia pediculicola sequences and the ecological form (head vs. body lice) or geographical origin, as similar symbiont haplotypes were distributed across human lice collected from various regions. Further global analysis of 16S‐rRNA revealed that these sequences belonged to the supergroup F of *Wolbachia*. These findings expand our understanding of *Candidatus* Riesia pediculicola, *Candidatus* Riesia pthiripubis and *Wolbachia* endosymbionts in human lice, and their potential applications in the evolutionary study and biological control of these ectoparasites.

## Author Contributions

Conceptualization: A.M., A.I., and M.A. Methodology: A.M. and M.A. Investigation: A.M., A.I., and M.A. Writing – original draft preparation: A.M. and M.A. Writing – review and editing: S.B., A.I., and M.A. All authors have read and agreed to the published version of the manuscript.

## Funding

The authors have nothing to report.

## Ethics Statement

Ethical approval for this study was granted through protocol number 95/99/AVC/ESA by the Avicenne Hospital Research Ethics Committee.

## Consent

A written consent was provided and signed by the parents including the authorisation for publishing the clinical information.

## Conflicts of Interest

The authors declare no conflicts of interest.

## Supporting information


**Data S1:** Supporting information.

## Data Availability

Data sharing not applicable to this article as no datasets were generated or analysed during the current study.
