# Supplementary material for: Detection and Genetic Diversity of Heritable Bacterial Symbionts in Human Lice Based on 16S‐rRNA Gene
Source: Environ Microbiol Rep. 2026 Jan 8;18(1):e70243. doi: 10.1111/1758-2229.70243 (PMC12784420; doi:10.1111/1758-2229.70243)
Supplement: Supplementary file 1 — Data S1: Supporting information. [file EMI4-18-e70243-s001.zip › SI.V.docx]

| **Table SI.V**. A summarized overview of the bacterial endosymbionts of human lice reported in the literature | | | | | |
| --- | --- | --- | --- | --- | --- |
| Author(s) | Lice species (N° of examined specimens) | Origin (country)* | Symbiont species (prevalence%) | | |
|  |  |  | *Candidatus* Riesia pediculicola | *Candidatus* Riesia pthiripubis | *Wolbachia* |
| Perotti et al. 2004 | Head lice (143) | 1 | - | - | Symbiont detection * |
| Kyei-Poku et al. 2005 | Head lice (undetermined) | 2 | - | - | Symbiont detection * |
| Covacin and Barker 2006 | Head, body & pubic lice (undetermined) | 4 | - | - | Symbiont detection * |
| Sasaki-Fukatsu et al. 2006 | Head (9) & body (57) lice | 2 | 100 | - | - |
| Allen et al. 2007 | Head (2), body (2) & pubic (1) lice | 3 | Symbiont detection * | Symbiont detection * | - |
| Perotti et al. 2007 | Head & body lice (undetermined) | 2 | Symbiont detection * | - | Symbiont detection * |
| Allen et al. 2016 | Head & body lice (undetermined) | 1 | Symbiont detection * | - | - |
| Boyd et al. 2017 | Head & body lice (undetermined) | 4 | Symbiont detection * | - | - |
| Dzul-Rosado et al. 2022 | Head lice (28) | 1 | - | - | 71.4 |
| Hammoud et al. 2022 | Head & body lice (73) | 12 | 78.1-89 | - | - |
| Boyd et al. 2024 | Head lice (446) | 43 | 53.6 | - | - |
| Present study | Head (186), body (11) & pubic (12) lice | 18 | Head (79.6) & body (81.8) lice | 41.7 | Head (95.2), body (100) & pubic (75) lice |

*: Symbiont detection without indicating accurate prevalence rate
